# In vitro binding and survival assays of *Leishmania *parasites to peripherical blood monocytes and monocyte-derived macrophages isolated from dogs naturally and experimentally infected with *Leishmania (Leishmania) chagasi*

**DOI:** 10.1186/1746-6148-3-11

**Published:** 2007-05-30

**Authors:** Weverton M Sampaio, Eliane P Moura, Felipe CS Arruda, Raul R Ribeiro, Cíntia F Alves, Ferdinan A Melo, Ana Paula SM Fernandes, Marilene SM Michalick, Maria N Melo, Washington L Tafuri, Wagner L Tafuri

**Affiliations:** 1 Departamento de Anatomia Patológica, Faculdade de Medicina, Universidade Federal de Minas Gerais, Belo Horizonte, MG, Av Alfredo Balena 190, CEP 30130-100, Brazil; 2 Departamento de Parasitologia, Instituto de Ciências Biológicas, Universidade Federal de Minas Gerais, Belo Horizonte, MG, Av Antonio Carlos 6627, CEP31270-901, Brazil; 3 Departamento de Análises Clínicas e Toxicológicas, Faculdade de Farmácia, Universidade Federal de Minas Gerais, Belo Horizonte, MG, Av Antonio Carlos 6627, CEP31270-901, Brazil; 4 Departamento de Patologia Geral, Instituto de Ciências Biológicas, Universidade Federal de Minas Gerais, Belo Horizonte, MG, Av Antonio Carlos 6627, CEP31270-901, Brazil

## Abstract

**Background:**

There are a few works considering the characterization of canine monocyte-derived macrophages as well as a standardized procedure for isolation, culture, and infection of these cells with *Leishmania*. We have performed several modifications in order to improve the canine monocyte-derived macrophage cultures. In addition, we have done a comparative study between monocytes and monocyte-derived macrophages from dogs naturally and experimentally infected with *L. chagasi*.

**Results:**

In the presence of exogenous serum, opsonized *Leishmania *promastigotes binds better to monocytes/macrophages than without serum. Otherwise, this binding occurs due to the strict correlation between the opsonized biologic particles with the third receptor of the complement (CR3-CD11b/CD18). In fact, our assays with CD11b confirmed the importance of this receptor for canine cells and the *L. chagasi *experimental system. Moreover, monocytes obtained from naturally infected dogs have shown a higher number of monocytes bounded to promastigotes. The experimental results regarding survival have shown that promastigote forms of opsonized *L. chagasi *were more infective, because we found higher numbers of promastigotes bound to the different cells. As a consequence, after forty-eight hours of binding, higher numbers of amastigotes appeared inside monocyte-macrophages.

**Conclusion:**

These studies have given support to continue comparative studies involving canine monocytes, monocyte-derived macrophages and peritoneal macrophages. Since we have standardized the canine cell culture, we are looking forward to determining the phenotypic properties of these cells before and after *L. chagasi *infection using flow cytometry.

## Background

Canine visceral leishmaniasis (CVL) is caused by *Leishmania infantum *(syn. *L. chagasi *in America) [1]. This protozoan is transmitted by the bite of the female phlebotomine sand fly of the species *Lutzomyia longipalpis *(in America) [2]. Protozoa of the genus *Leishmania *are dimorphic obligate intracellular parasites that reside within mononuclear phagocytes in the mammalian host. In America, dogs infected with *L. chagasi *constitute the main domestic reservoir of the parasite and play an important role in transmission to humans, in which the parasite causes the visceral disease, characterizing it as a zoonosis.

The immune response against *Leishmania *is highly dependent upon macrophages. Although these are the host cells targeted for infection, they are competent to present antigen and kill intracellular *Leishmania *[3]. Isolation, culture and characterization of alveolar and peritoneal macrophages [4], bone marrow-derived macrophages [5], and canine macrophage cell lines derived from malignant histiocytosis [6] have been described. Also, the interaction of *Leishmania *promastigotes with mononuclear phagocytes has been characterized, and multiple receptor-ligand interactions have been implicated in the attachment to, and uptake of, promastigotes by macrophages [7]. These interactions include the binding of parasite surface molecules (lipophosphoglycan and gp63) or host-derived opsonins (complement, fibronectin and immunoglobulin) to multiple macrophage receptors.

Many *in vitro *methods for investigating the *Leishmania-*macrophage interaction have been described in the literature. These techniques involve quantifying parasites and macrophages on coverslips, where parasites can be counted by conventional microscopy, immunofluorescent microscopy (immunolabelled parasites) or radioactivity associated with the cell lysates [8-10]. However, these techniques are laborious and time-consuming and do not allow the cells and surface receptors involved in parasite uptake to be characterized accurately. Moreover, most of these studies have involved human or murine monocytes/macrophages and *Leishmania major*. Few *in vitro *studies using canine macrophages and *Leishmania chagasi *have been published. As far as we know, Madeira et al. (1999) [11] were the first to assay binding between different species of *Leishmania *and canine macrophages. Recently, Gonçalves et al. (2005) [12] developed a novel method using flow cytometry and canine peritoneal macrophages to study this interaction, and Bueno et al. (2005) [13] described a phenotypic, functional and quantitative characterization of canine peripheral blood monocyte-derived macrophages. The cells in these studies were inoculated with *L. chagasi *promastigotes to assess their phagocytic activity.

There have been few studies characterizing canine monocyte-derived macrophages and employing a standardized procedure for isolation, culture and infection of these cells with *Leishmania*. To date, as described by Ho & Babiuk (1979) [14] and Bueno et al. (2005) [13] (personal communication), our own experience has shown that the number of monocytes that can be isolated from dog blood is usually low, especially for obtaining monocyte-derived macrophage cultures. In this study, therefore, we have introduced several modifications to improve canine monocyte-derived macrophage cultures. We have also compared monocytes and monocyte-derived macrophages from dogs naturally and experimentally infected with *L. chagasi*.

## Results

### The binding of *L. chagasi *promastigotes to monocytes as determined by optical microscopy

**Figure 1:**
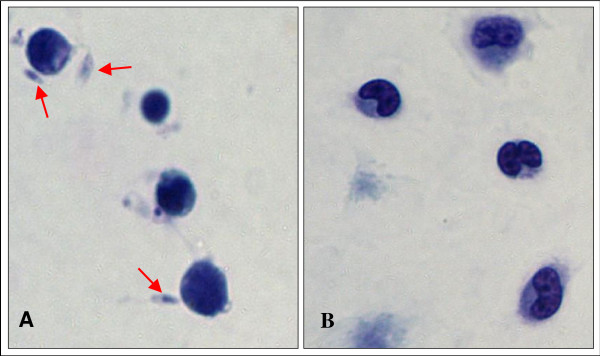
**The binding of *Leishmania *promastigotes to canine monocytes**. (A) Promastigotes binding to peripherical monocytes from one animal naturally infected (ANI) with *Leishmania chagasi *in the presence of C5D serum. (arrows) (B) Only monocytes can be observed over coverslips (assay with absence of serum C5D). Giemsa. 1000×.

**Figure 2:**
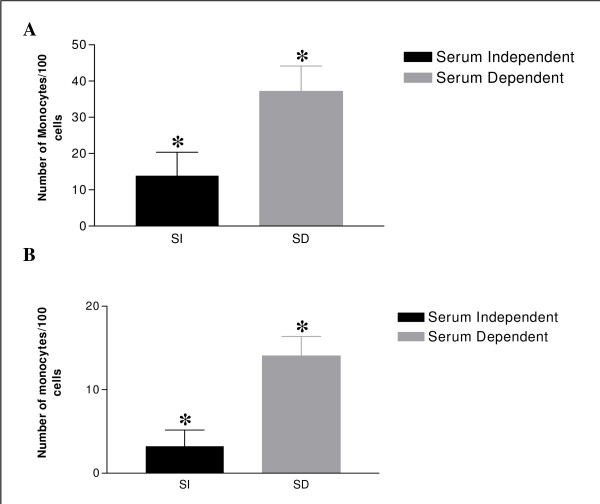
**The binding of *Leishmania *promastigotes to canine monocytes**. (A) Average of *Leishmania *promastigotes bound to monocytes of animals naturally infected (ANI) with *L. chagasi *in the presence or absence of C5D serum (2.5 × 10^6 ^parasites/well) (* p < 0.01) (B) Average of *Leishmania *promastigotes bound to monocytes of animals experimentally infected (AEI) with *L. chagasi *in the presence or absence of C5D serum (2,5 × 10^6 ^parasites/well) (*p < 0.01).

By microscopy, we easily observed the adhesion of *Leishmania *promastigotes to monocytes in the presence of C5D serum (Figure 1(a). This result was statistically significant (p < 0.01) and occurred in both groups of infected animals, as depicted in Figure 2(a), 2(b). The adhesion was independent of the concentration of promastigotes.

**Figure 3:**
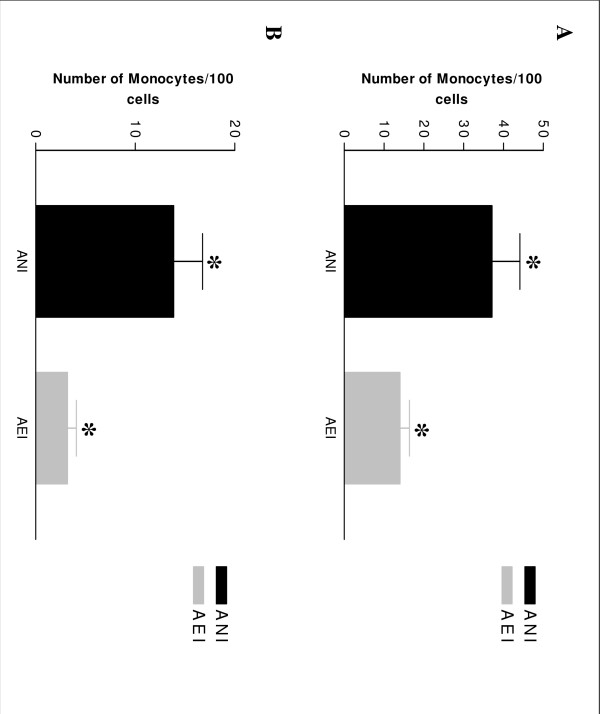
**The binding of *Leishmania *promastigotes to canine monocytes**. (A) to *Leishmania *promastigotes bound to monocytes from animals naturally infected (ANI) with *L. chagasi *and animals experimentally infected (AEI) with *L. chagasi *in the presence of C5D serum (2.5 × 10^6 ^parasites/well) (*) p < 0.01. (B) *Leishmania *promastigotes bound to monocytes from dogs naturally and experimental infected with *L. chagasi *in the absence of C5D serum (2.5 × 10^6 ^parasites/well) (*) p < 0.01.

Interestingly, significantly more monocytes from naturally-infected animals than from experimentally-infected ones were bound to *Leishmania *promastigotes (p < 0.01) (Figure 3). This was also independent of the promastigote concentration and of the presence or absence of C5D serum. However, in the presence of C5D serum, the average number of *Leishmania *promastigotes bound to monocytes (100 cells/well) was 1.67 for naturally-infected and 0.32 for experimentally-infected dogs. In the absence of serum, these averages decreased to 0.55 and 0.05, respectively.

### The binding of *L. chagasi *promastigotes to monocytes as shown by flow cytometry

Aliquots of CFSE-stained promastigotes were evaluated using the flow cytometer to analyze the efficiency of CFSE staining. More than 95% were strongly and homogeneously stained with CFSE, and unstained promastigotes were easily distinguished as previously described [12].

The cells were run on an analytical flow cytometer equipped with a laser emitting at 488 nm (FACSVantage, Becton & Dickinson, San José, CA, USA). Whole cells were distinguished from fragments by gating based on the forward and side scatter signals. The receptor monocytes were detected by their fluorescence intensities relative to those of uninfected cells using the FL2 (red) and FL3 (brown) detectors. Both the frequency and the intensity of receptors associated with cells were determined. Analyses were performed on 30,000 gate events, and numerical data were processed using WinMDI software version 2.8 [15].

**Figure 4:**
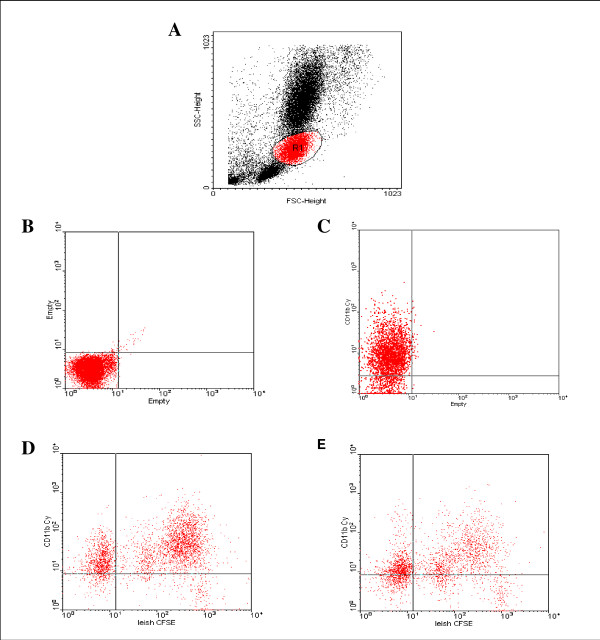
**Flow cytometer analysis of CD11b integrin expression and the binding of *Leishmania *promastigotes to canine monocytes**. (A) Gate R1 was built separating monocytes from animals naturally infected (ANI) with *L. chagasi *by cells size (x axis) and granularity (y axis). (B) Gate with mononuclear cells (control) (C) CD11b positive cells; (D) CD11b expression during the *L. chagasi *binding to mononuclear cells with the presence of C5D serum. (*E*) CD11b expression during the *L. chagasi *binding to mononuclear cells with the absence of C5D serum.

**Figure 5:**
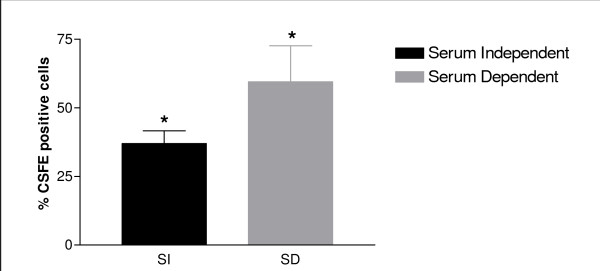
**Flow cytometer analysis of the binding of *Leishmania *promastigotes to canine monocytes**. Serum dependent conditions induce a higher association of *Leishmania *with monocytes as measured by both mean fluorescent intensity (CFSE) and percentage of monocytes associated with *Leishmania*. (*) p < 0.05.

Figures 4(a) and 4(b) show the gate that was built to separate monocytes by size (x axis) and granularity (y axis) from dogs naturally infected with *L. chagasi*. Figure 4C depicts CD11b-positive monocytes (72.61%). Figures 4(d) and 4(e) show CD11b expression on monocytes 50 min after the start of the interaction with CFSE-stained *L. chagasi *in the presence and absence of C5D serum, respectively. The percentage of CD11b-positive monocytes decreased to 19.26% in the presence of serum; in the absence of serum it was 42.25%. These data indicate that the expression of CD11b receptors decreased in the presence of serum because of the opsonized *Leishmania *promastigotes. The percentages of CSFE-*Leishmania *positive monocytes from serum-dependent and serum-independent cultures were 59.50% and 36,98%, respectively. This means that the serum enhanced *Leishmania *binding to the monocytes (Figure 5).

### Canine peritoneal macrophages and monocyte-derived macrophages

**Figure 6:**
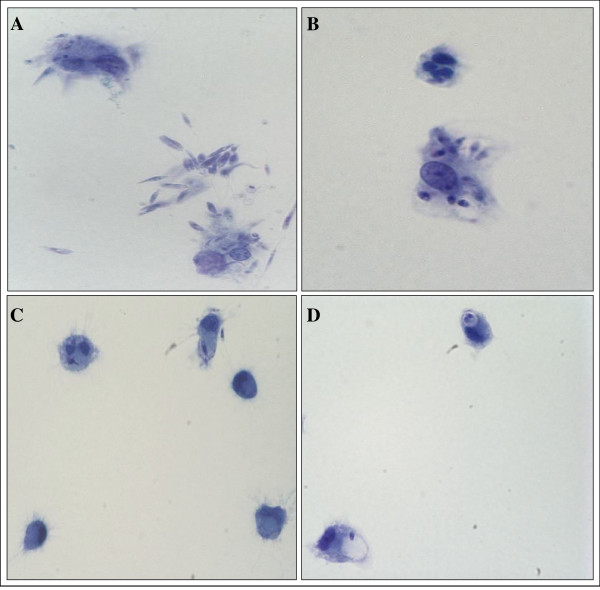
**The binding and survival assays of *Leishmania *promastigotes to monocytes-derived macrophages**. (A) Promastigotas adhesion fifty minutes after incubation with the presence of C5D serum. (B) Note many amastigotes into could be seen 48 hours after interaction with the presence of C5D serum. (C) Promastigotas adhesion with the absence of serum. (D) Note some amastigotas could be seen 48 hours after interaction with the absence of C5D serum Giemsa. 1000×.

**Figure 7:**
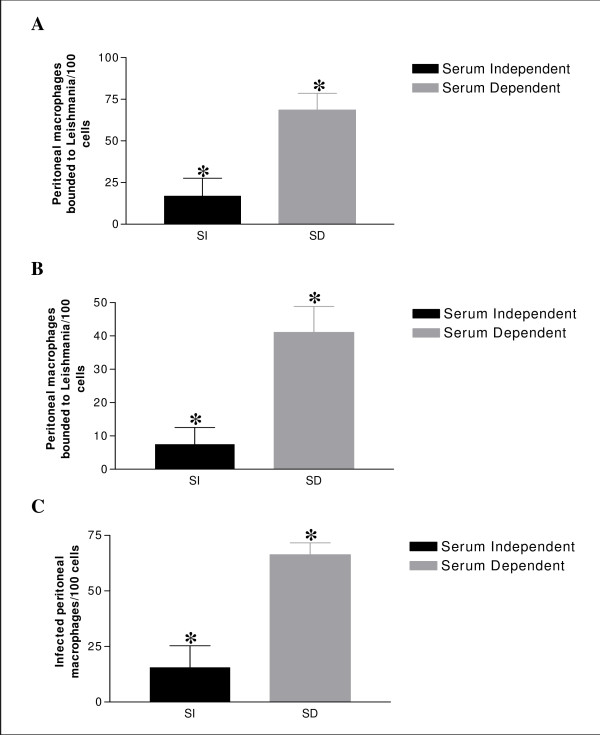
**The binding and survival assays of *Leishmania *promastigotes to peritoneal macrophages**. (A) Average of peritoneal macrophages binding to *Leishmania *from naturally infected animals with the presence or absence of C5D serum, after 50 min incubation (2,5 × 10^6 ^parasites/well) (B) Average of peritoneal macrophages binding to *Leishmania *from experimental infected animals with the presence or absence of C5D serum, after 50 min incubation (2,5 × 10^6 ^parasites/well). (C) Average of infected peritoneal macrophages binding to *Leishmania *from naturally infected animals with the presence or absence of C5D serum after, fourth eight hour incubation (5 × 10^6 ^parasites/well) (*) p < 0.01.

**Figure 8:**
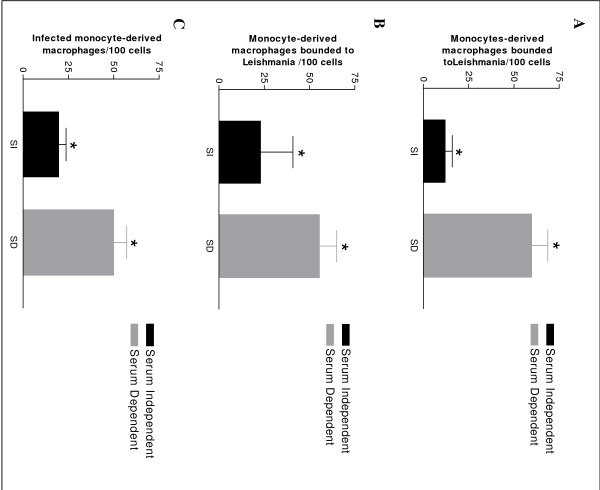
**The binding and survival assays of *Leishmania *promastigotes to monocyte-derived macrophages**. (A) Average of monocyte-derived macrophages binding to *Leishmania *from naturally infected animals with the presence or absence of C5D serum, after 50 min incubation (5 × 10^6 ^parasites/well) (B) Average of monocyte-derived macrophages binding to *Leishmania *from experimental infected animals with the presence or absence of C5D serum, after 50 min incubation (5 × 10^6 ^parasites/well). (C) Average of infected peritoneal macrophages binding to *Leishmania *from experimental infected animals with the presence or absence of C5D serum after, fourth eight hour incubation (5 × 10^6 ^parasites/well) (*) p < 0.01.

The numbers of peritoneal macrophages and monocyte-derived macrophages bound to *Leishmania *promastigotes were significantly increased when C5D serum was used during the interaction with the canine cells. Moreover, parasite survival, characterized by the presence of amastigote forms of *Leishmania *in macrophages, was also significantly increased in the presence of serum (p < 0.01) (Figure 6). These results were found in both the naturally and the experimentally infected animals and were independent of incubation time (50 min or 48 h) and parasite concentration (Figures 7, 8) (p < 0.01). However, we observed no difference between the naturally and the experimentally infected dogs. As with the peripheral blood monocytes from naturally-infected dogs, significantly more cells were bound to *Leishmania*.

## Discussion

There is an extensive literature on macrophage physiology and function in laboratory animals and humans. Several groups have reported characterizations of canine macrophage populations, including bone marrow-derived macrophages [5], peripheral blood monocytes [14,16] and canine macrophage cell lines derived from malignant histiocytosis [6]. However, few studies have concerned the isolation and culture of canine monocytes and monocyte-derived macrophages [17]. The procedures for obtaining these cells are not simple and are less convenient than obtaining cells from blood, although the number of monocytes that can be isolated from blood is usually relatively low [14]. Moreover, cultures of monocyte-derived macrophages are not easy to maintain in the laboratory. Bueno et al. (2005) [13] demonstrated a non-significant negative correlation between the number of monocyte-derived macrophages after 10 days in culture and the number of monocytes in the original blood sample. These authors concluded that the number of monocytes in a given blood sample is not predictive of the macrophage yield after culture. However, flow cytometric analysis of the Teflon-adherent cells after ten days in culture revealed that monocyte-derived macrophages are phenotypically and functionally compatible with macrophages, and are larger and more granular than monocytes and lymphocytes.

In this work we have followed the method employed by Bueno et al (2005) [13], modified by supplementing the medium with GMCSF and autologous canine serum [18]. These modifications improved the survival of cell monolayers *in vitro*. Thus, we were able to obtain viable canine cell cultures with better morphology and sufficient numbers of cells for all the experiments. In addition, the canine cell cultures were maintained for a long time so that it was possible to carry out survival assays. Thus, on the basis of this culture standardization, we developed assays for comparing monocytes, monocyte-derived macrophages and peritoneal macrophages experimentally using the parasite *Leishmania chagasi*.

Our results are comparable to those obtained by other *in vitro *studies with monocytes/macrophages and *L. majo*r. Opsonized *Leishmania *promastigotes bind better to monocytes/macrophages in the presence of exogenous serum than they do in its absence [19,20]. Otherwise, this binding depends on the strict correspondence between the opsonized biological particles and the third complement receptor (CR3-CD11b/CD18) [9,18]. Our CD11b assays confirmed the importance of this receptor for the canine cell-*L. chagasi *system. Gonçalves et al. (2005) [12] previously described the importance of working with peritoneal macrophages from dogs naturally infected with *L. chagasi*. Now we have found similar results using peripheral monocytes.

We found an interesting result with peripheral blood monocytes and *Leishmania *promastigotes 50 min after the beginning of the interaction (binding assay): more promastigotes were bound to monocytes obtained from naturally-infected than from experimentally-infected dogs. These results could be consistent with the observation that amastigote forms of *Leishmania *are more easily found in tissues (for example, livers and spleens) from naturally-infected than from experimentally-infected dogs (data not shown).

The experimental results regarding survival showed that promastigote forms of opsonized *L. chagasi *were more infective, because we found more promastigotes bound to the different cells. In consequence, after 48 h of binding, more amastigotes appeared inside the monocytes/macrophages. This is in accordance with Mosser & Edelson (1985) [19], who used murine macrophages and *L. major *promastigotes. They showed that opsonized parasites are more infective, thereby improving the survival of amastigotes in macrophages. These findings will support continuing comparative studies involving canine monocytes, monocyte-derived macrophages and peritoneal macrophages. Since we have standardized the canine cell culture, we will be able to determine the phenotypic properties of these cells before and after *L. chagasi *infection using flow cytometry.

## Methods

### I – Animals

#### Animals naturally infected (ANI) with *L. chagasi*

Five mongrel dogs of unknown age were obtained from the City of Santa Luzia (suburban area of Belo Horizonte, City Hall Zoonosis Department), MG, Brazil. All dogs were positive for *Leishmania *by indirect immunofluorescence (IIF), complement fixation tests (CFT) and ELISA (enzyme-linked immunosorbent assay). Animals were maintained with food and water "ad libitum". All animal studies were performed under the guidance and approval of the institute's animal welfare committee under the supervision of a certified veterinarian. The experimental protocol using dogs was approved by CETEA (Comitê de ética em experimentação animal – UFMG), number 034/2004.

#### Preparing parasites for canine experimental infection

Promastigote forms of *Leishmania (Leishmania) chagasi *strain MHOM/BR/1972/BH6 were maintained in hamsters and cultivated *in vitro *with NNN/MEM (Gibco – BRL – USA) with 10% inactivated fetal calf serum (FCS) (Cultilab – SP – BR) supplemented with 2 mM glutamine, 100 UI/ml penicillin G and 100 μg/ml streptomycin, and maintained at 23°C in a FANEM^® ^incubator (model 347). The *Leishmania *promastigotes in stationary phase were washed once with phosphate buffered saline (PBS), centrifuged at 200 g for 10 min/18°C and resuspended to 1 × 10^7 ^parasites/ml in PBS.

#### Experimental canine infection

Experimental infection was carried out at HERTAPE CALIER SAÚDE ANIMAL S/A (Juatuba, MG). A group of 3-month-old laboratory-bred beagles (3 males and 2 females) were inoculated intravenously (saphenous vein) with 1 × 10^7 ^promastigotes/ml resuspended in PBS. The infection was confirmed parasitologically and seroconversion was evident after 90 days post-infection. A second group of 5-month-old male *L. chagasi *seronegative beagles that served as uninfected controls were inoculated with sterile PBS at the time of *L. chagasi *inoculation of the experimentally infected animals (AEI).

### II – *In vitro *assays

#### Obtaining canine monocytes and monocyte-derived macrophages

Peripheral blood (120 ml) was collected from the external jugular vein into heparanized tubes. To isolate peripheral blood mononuclear cells (PBMC), the blood was centrifuged at 300 g for 10 min at room temperature. Plasma was separated and blood cells were resuspended in PBS (1:1 proportion), overlaid on Ficoll solution (HISTOPAQUE 1077 – SIGMA) at a ratio of 1 Ficoll:2 blood, and centrifuged at 200 g for 40 min at 4°C. PBMC were separated, resuspended and washed twice in PBS (1:1 proportion), centrifuging at 300 g for 10 min at 4°C. They were resuspended in 8 ml RPMI-1640 (GIBCO, CARLSBAD, US) supplemented with 10% FCS (CUTILAB), 200 mM L-glutamine, 10 mM pyruvate, 10 mM non-essential amino acids, 7.5% w/v sodium bicarbonate (Gibco, Carlsbad, USA), 50 IU/ml penicillin and 50 μl/100 ml streptomycin. This medium was adjusted to pH 7.4.

In order to obtain monocyte-derived macrophages, another part of the peripheral blood suspension was transferred to Teflon flasks (NalgeNunc, Rochester, USA), supplemented with 10% autologous canine serum and 20% DE GMCSF (granulocyte-macrophage colony-stimulating factor), and cultured at 37°C with 5% CO_2 _(Forma Scientific Incubator, Waltham, USA). The medium was changed to remove non-adherent cells 24 h later and the culture was maintained under the same conditions for 10 days, changing the medium every 3 days. After 10 days in culture [21] the cells formed a monolayer, as observed by phase contrast microscopy. The macrophage-containing Teflon flasks were placed on ice for 30 min and agitated to harvest the cells. The cells were washed with cold PBS, as described by Bueno et al. (2005) [13] with modifications: we supplemented the medium with GMCSF obtained from L-cells (mouse fibroblasts) and autologous canine serum [18]. The cells were then were transferred to falcon tubes (50 ml), centrifuged at 600 g for 15 min and resuspended in 1 ml complete RPMI-1640. The concentration of the suspension was adjusted to 3 × 10^6 ^viable cells/ml and counted in a Newbauwer chamber.

#### Obtaining canine peritoneal macrophages

To obtain the peritoneal macrophages, the dogs were anesthetized with 2.5% Thiopental sodium (1 ml/kg) and sacrificed using a lethal dose (0.3 ml/Kg) of T-61^® ^(Intervet). The animals were positioned in decumbency and the peritoneal cavity was disinfected using iodine-alcohol and washed with sterile saline, as described by Gonçalves et al. (2005) [12]. The washed peritoneal cell suspensions were adjusted to 3 × 10^6 ^cells/ml in culture medium (D-10 - DMM + 10% FCS + L-glutamine + penicillin-streptomycin).

#### Preparing parasites for assays for binding and survival in monocyte-macrophages and peritoneal macrophages

*Leishmania (Leishmania) chagasi *parasites (stationary phase – MHOM/BR/1972/BH6) were adjusted to 5 × 10^7 ^cells/ml in "phagocytosis buffer" (PB) culture medium (equal parts of Dulbecco's Modified Eagle Medium and Medium 199 supplemented with 1% BSA and 12.5 mM HEPES) [18].

#### Leishmania binding assays

The interactions between *Leishmania chagasi *promastigotesand canine peritoneal monocytes, monocyte-derived macrophages and peritoneal macrophages were measured as follows. Cells were added to 24-well plates containing 3 × 10^5 ^cells/100 μl. All binding assays were performed in triplicate in a PB consisting of equal parts of medium 199 and Dulbecco's modified Eagle medium (Mediatech) supplemented with 1% BSA [18]. Assays performed in the presence of serum were carried out at a concentration of 5% of AKRJ C5-deficient mouse serum (C5D) [22] with a final volume of 400 μl in each well.

The use of C5D serum obviates any problems associated with complement-mediated lysis of the parasites [1]. Promastigote forms of *L. chagasi *(3 × 10^6 ^or 5 × 10^6^/well) were added to the monolayer cells. After 50 min incubation at 35°C, unbound promastigotes were removed by thorough washing. The monolayers and bound promastigotes were fixed with 2.5% glutaraldehyde and stained with 10% Giemsa. The number of promastigotes bound per well was deduced using an optical microscope, assuming 100 monocytes-macrophages/well. All the experiments were done in triplicate [20].

#### Survival assay

Assays to measure the internalization of promastigotes into canine monocyte-derived macrophages and peritoneal macrophages and visualization of amastigotes were performed in a similar way to the binding assays after the *Leishmania *had interacted with the cells for 48 h. The monolayers with intracytoplasmatic amastigotes in the macrophages were fixed with 2.5% glutaraldehyde and stained with 10% Giemsa. The number of parasitized macrophages and intracellular amastigotes was deduced by optical microscopy, assuming 100 macrophages/well. All experiments were done in triplicate [20].

#### Staining of parasites and binding assay for flow Cytometric analysis

Promastigote forms of *Leishmania *were resuspended in 1 ml PBS (5 × 10^7 ^parasites/ml) with 1 μl of CFSE (carboxyfluorescein diacetate succinimidyl ester – Molecular Probes C-1157) from a 2.8 μg/ml stock in DMSO (dimethyl sulfoxide), and incubated in a 37°C water bath for 10 min in the dark as described by Gonçalves et al. (2005) [12]. The CFSE-stained *Leishmania chagasi *promastigotes were allowed to interact with canine cells as follows: (1) monocytes obtained from five dogs naturally infected with *L. chagasi*; (2) peritoneal macrophages from three naturally-infected dogs and one experimentally-infected dog. These interactions took place in polypropylene tubes to avoid cell adhesion. Canine cells (100 μl, 3 × 10^5 ^cells) and 100 μl CFSE stained-*Leishmania chagasi *(5 × 10^6 ^cells) were combined in the tubes and maintained for 45–60 min at 37°C in a 5% CO_2 _atmosphere. The assays were performed in the presence of either normal serum or a final concentration of 5% C5-deficient mouse serum (from AKR/J mice) [22].

#### Specific staining with anti-CR3

Canine cells obtained as described above were stained with anti-CR3 (CD11b/CD18) rat anti-human CD18-RPE antibody (Serotec), which cross-reacts with canine cells. Nonconjugated, purified mouse anti-canine CD11b (Serotec) was conjugated using a Zenon tricolor kit (Molecular Probes – Z-25080), as described by the manufacturer, with the "C" compound that represents a 647 nm emission band and can be used with CFSE and R Phycoerythrin (RPE). The cells were incubated with labeled antibody solutions for 20 min at 4°C. After staining, the preparations were washed with 0.1% sodium azide in PBS, fixed with 200 μl 2% formaldehyde in PBS and kept at 4°C until data were acquired by flow cytometry (FACSVantage, Becton & Dickinson, San José, CA, USA).

#### Binding analysis by flow cytometry

The cells were run on an analytical flow cytometer equipped with a laser emitting at 488 nm (FACSVantage, Becton-Dickinson, San Diego, CA, USA). Whole cells were distinguished from fragments by gating based on the forward and side scatter signals. CFSE-stained promastigotes bound to macrophages were identified by their fluorescence intensities relative to those of uninfected cells using the FL1 (green) detector. Both the frequency of cells associated with promastigote forms of *Leishmania *and the intensity of cells associated with *Leishmania *were determined as described previously [12].

### Statistical analysis

Results are given as a complete randomized design and the means from each group were compared using Student's t test; p values less than 0.05 were considered significant. All analyses were carried out using Prism 3.0 software.

## Authors' contributions

WMS; RRR, EPM, CFA, WLT: Carried out the necropsies

WMS, FCSA, EPM: Carried out the *in vitro *assays

MNM, CFA: Carried out all the *Leishmania chagasi *cultures

WMS, RRR: Carried out the statistical analysis

MSMM, RRR: Carried out the serological tests [CRF, ELISA, IFAT, TRALDd] for *Leishmania infection*

APSMF, WLT, WLT: Carried out the canine experimental infection with *L. chagasi*

WMS, WLT, WLT have made substantial contributions to conception and design, analysis and interpretation of data
